# Influence of the RDL A301S mutation in the brown planthopper *Nilaparvata lugens* on the activity of phenylpyrazole insecticides

**DOI:** 10.1016/j.pestbp.2017.01.007

**Published:** 2017-10

**Authors:** William T. Garrood, Christoph T. Zimmer, Oliver Gutbrod, Bettina Lüke, Martin S. Williamson, Chris Bass, Ralf Nauen, T.G. Emyr Davies

**Affiliations:** a Biological Chemistry and Crop Protection Department, Rothamsted Research, Harpenden, Hertfordshire AL5 2JQ, UK; b College of Life and Environmental Sciences, Biosciences, University of Exeter, Penryn Campus, Penryn, Cornwall TR10 9FE, UK; c Bayer CropScience AG, R&D, Research Technologies, Monheim, Germany; d Bayer CropScience AG, R&D, Pest Control Biology, Monheim, Germany

**Keywords:** Ethiprole, RDL, A301S, Q359E, Insecticide resistance

## Abstract

We discovered the A301S mutation in the RDL GABA-gated chloride channel of fiprole resistant rice brown planthopper, *Nilaparvata lugens* populations by DNA sequencing and SNP calling via RNASeq. Ethiprole selection of two field *N. lugens* populations resulted in strong resistance to both ethiprole and fipronil and resulted in fixation of the A301S mutation, as well as the emergence of another mutation, Q359E in one of the selected strains. To analyse the roles of these mutations in resistance to phenylpyrazoles, three *Rdl* constructs: wild type, A301S and A301S + Q359E were expressed in *Xenopus laevis* oocytes and assessed for their sensitivity to ethiprole and fipronil using two-electrode voltage-clamp electrophysiology. Neither of the mutant *Rdl* subtypes significantly reduced the antagonistic action of fipronil, however there was a significant reduction in response to ethiprole in the two mutated subtypes compared with the wild type. Bioassays with a *Drosophila melanogaster* strain carrying the A301S mutation showed strong resistance to ethiprole but not fipronil compared to a strain without this mutation, thus further supporting a causal role for the A301S mutation in resistance to ethiprole. Homology modelling of the *N. lugens* RDL channel did not suggest implications of Q359E for fiprole binding in contrast to A301S located in transmembrane domain M2 forming the channel pore. Synergist bioassays provided no evidence of a role for cytochrome P450s in *N. lugens* resistance to fipronil and the molecular basis of resistance to this compound remains unknown. In summary this study provides strong evidence that target-site resistance underlies widespread ethiprole resistance in *N. lugens* populations.

## Highlights

• Ethiprole selection of *N. lugens* increases resistance to ethiprole and fipronil.
• Ethiprole selection increases frequency of the RDL A301S and a new mutation, Q359E.
• The A301S mutation, but not Q359E, confers significant resistance to ethiprole.
• Neither A301S or Q359E confer significant resistance to fipronil.

## Graphical abstract

**Figure:**
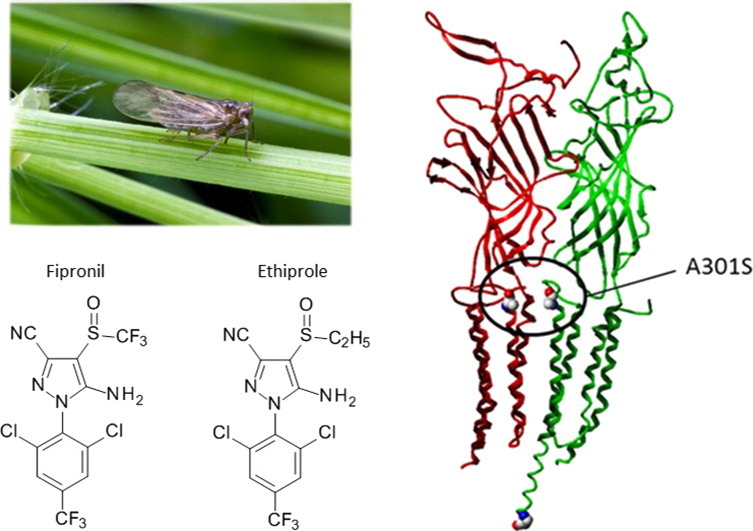

## 1 Introduction

The brown planthopper (BPH), *Nilaparvata lugens* Stål (Hemiptera: Delphacidae), is a key economic pest of rice (*Oryza sativa* L.) throughout Asia. It is a monophagous herbivore and affects the rice crop through direct feeding causing nutrient depletion in the plant. This causes a series of deleterious effects that leads to ‘hopperburn’, which is characterised by visible stunting, wilting and browning of the affected crop. BPH is also an efficient vector for various rice viruses, including ragged rice stunt and grassy stunt virus [1]. These combined can cause significant damage to rice crops, with up to 60% loss of yield in susceptible cultivars [2].

The application of chemical insecticides has been the preferred method to control BPH, however, this has inevitably led to the evolution of resistance and a reduction in effectiveness. Resistance has affected many of the major classes of insecticides including organophosphates, carbamates, pyrethroids, neonicotinoids and phenylpyrazoles [3], [4], [5], [6]. Understanding the levels of resistance through monitoring and analysing the mechanisms responsible for this resistance is a core concept behind being able to effectively control BPH through resistance management strategies.

The phenylpyrazole (fiprole) insecticides, such as ethiprole and fipronil were introduced for BPH control after resistance to imidacloprid became commonplace [7]. Phenylpyrazoles are described as non-competitive blockers of the *gamma*-aminobutyric acid (GABA)-gated chloride channel, a member of the pentameric transmembrane cys-loop ligand-gated ion channel family mediating synapse inhibition in the insect central nervous system [8], [9], [10]. Fiproles are potent inhibitors of GABA-mediated inhibitory nerve transmission and belong to group 2 of the MoA classification scheme of the Insecticide Resistance Action Committee (IRAC), that encompasses GABA-gated chloride channel antagonists [11]. This MoA class also includes much older insecticide chemistry, such as the cyclodiene hydrochlorines, which include endosulfan and dieldrin [12]. Ethiprole is structurally similar to fipronil only differing in an ethylsulfinyl substituent replacing the trifluoromethylsulfinyl moiety in fipronil [13].

Structural change by replacements of alanine 301 in the GABA-gated chloride channel, encoded for by the *Rdl* (*Resistance to dieldrin*) gene, has been linked to high levels of resistance to insecticidal antagonists, in particular cyclodiene organochlorines [14]. The most common substitution at this position, A301S, was first identified in *Drosophila melanogaster* and shown to cause 4000-fold resistance to dieldrin [15]. However, the role of this mutation in resistance to the newer fiprole insecticides has been debated [16], [17]. Other mutations at this amino acid residue, situated in the M2 transmembrane domain, have also been associated with fipronil resistance. A 20,000-fold fipronil resistant strain of *Drosophila simulans* exhibited a A301G replacement at this position in combination with a substitution at a second site, T350M in the M3 domain [18]. Functional expression of *Rdl* in *Xenopus* oocytes showed that the A301G mutation has modest effects on fipronil action, while a receptor variant with both of the mutations exhibited higher levels of resistance to fipronil [18]. A third substitution at the A301 position, A301N (A2'N), has been recently associated with fipronil resistance in two other rice planthopper species, *Sogatella furcifera* and *Laodelphax striatellus* [19], [20]. In the former species the A301N mutation was identified in association with a R340Q mutation in the cytoplasmic loop between M3 and M4 of the *S. furcifera* RDL with membrane potential assays suggesting the influence of the double mutation on fipronil resistance was more profound than that of the A301N alone [21]. This finding parallels that of the earlier work in *Drosophila* suggesting two mutations in RDL, one at AA residue 301 and one elsewhere act in concert to influence the level of in vivo resistance to fipronil [16]. However, in contrast to these findings other electrophysiological in vitro studies have revealed no significant differences in fipronil antagonist potency between wildtype and A301S RDL variants expressed in *Xenopus* oocytes [22], [23].

Very recently the A301S mutation was also identified in *N. lugens* and correlated with low levels of resistance to fipronil (5-fold in the presence of enzyme inhibitors and 23-fold without) [24]. The authors of this study also identified a second substitution in TM2 (R299Q) that in combination with A301S, was associated with much higher levels of resistance in a laboratory selected strain (96-fold with synergists, 237-fold without). Expression of recombinant RDL receptors, showed the R299Q mutation has a profound effect on the normal functioning of the receptor in response to the endogenous agonist GABA, suggestive of a strong fitness cost. However, the deleterious effects of R299Q was reduced in the presence of the A301S mutation. Surprisingly, the R299Q substitution was identified at extremely low frequency in field populations of *N. lugens* suggesting this is not the main mechanism of resistance in field populations [24].

Due to the evolution of resistance to fipronil in populations of *N. lugens* throughout Asia, and potential issues with the environmental toxicity of this insecticide, most growers subsequently switched to using ethiprole [25], [26]. Unfortunately, the rapid uptake of this insecticide has led to recent reports of resistance [5]. To date, the molecular basis of resistance to this insecticide has not been characterised and the potential role of mutations in the GABA-receptor remain unexplored. Metabolic resistance has been implicated in an ethiprole resistant BPH field strain from Thailand [27], though the authors also speculated that GABA receptor mutations could play a role in ethiprole resistance. Another study implicated two cytochrome P450s, CYP4DE1 and CYP6CW3v2, in ethiprole resistance in L. *striatellus* [28].

The aim of this study was to screen the *Rdl* gene for potential mutations in phenylpyrazole resistant BPH field and laboratory selected strains. We report here on the identification of the A301S mutation and a novel mutation, Q359E, and examine their role in fiprole resistance in vivo and in vitro. The potency of these mutations in causing ethiprole resistance was further assessed in *D. melanogaster*.

## 2 Material and methods

### 2.1 *N. lugens* strains and laboratory selection

The laboratory maintained strain of *N. lugens* (Bayer-S) was provided by Bayer CropScience (Monheim, Germany). The field strains Nl33 (South Vietnam, collected November 2010) and Nl55 (East Godavari District, Andhra Pradesh, India, collected February 2012) were provided by Bayer CropScience. Nl33 and Nl55 demonstrated high levels of resistance to ethiprole and were then placed under further selection with ethiprole in the laboratory. Strains of Nl33 and Nl55 were reared on rice plants sprayed with successively higher concentrations (ranging between 7.5 and 100 mg L^− 1^) of ethiprole over 15 generations. A second culture of Nl33 and Nl55 was maintained on untreated rice plants. All strains were reared in the laboratory on whole rice plants (*O. sativa* L. ssp.) under controlled environmental conditions (26 °C, 16 h photoperiod and 70% relative humidity).

### 2.2 *D. melanogaster* strains

Fly strains utilised in this study were maintained on standard food (Bloomington formulation) at 24 °C. The wild type strain Canton-S (#1, wild type) and the A301S strain (#35492, *Rdl*^MD-RR^) were sourced from the Bloomington *Drosophila* Stock Center at Indiana University, USA.

### 2.3 Leaf dip bioassay

Adults were taken from age-structured populations and were aged < 10 days old. Rice stems (10 cm cut length) were dipped into the required concentrations of formulated fiprole insecticide (ethiprole SC 200 and fipronil WG 80, Bayer CropScience, Monheim, Germany) for 20 s, air-dried and placed in a plastic specimen tube. Approximately 15 adults were aspirated directly into each tube and sealed with a ventilated lid. A small hole (3 mm diameter) was drilled in the base of each of the tubes, which were then stored vertically in a water bath (submerging only the base of each stem) at 26 °C for 72 h. Mortality was assessed and adults showing no sign of movement were scored as dead. Bioassays consisted of 3 replicates at each concentration. For synergism assays, each insect was treated upon the pronotum with 0.2 μL of 100 mg/L^− 1^ piperonyl butoxide (PBO in acetone) (20 ng adult^− 1^) and then transferred to rice stems dipped in fipronil. Mortality was assessed at 48 h.

### 2.4 Genotyping via sequencing

Genomic DNA from individual adults was extracted using 15 μl microlysis plus extraction buffer (Microzone Ltd., Haywards Heath, Sussex, UK) following the manufacturer's recommended protocol for tough cells. A typical PCR (25 μl) contained 0.5 μM of each primer (Table S1), 2 μl extracted DNA, 12.5 μl DreamTaq (Thermo Fisher, Waltham, MA, USA) containing Taq polymerase, 2 × PCR buffer and 4 mM MgCl_2_ (2 mM final concentration). Cycling conditions were 95 °C for 2 min followed by 30 cycles of 95 °C for 30 s, 57 °C for 30 s and 72 °C for 1 min, and a final elongation at 72 °C for 5 min. PCR products was verified by agarose gel electrophoresis prior to PCR cleanup and sequencing which was carried out by Eurofins Genomics (Ebersberg, Germany). Sequence analysis and protein alignments were done with Geneious R8 (Biomatters, Auckland, New Zealand).

### 2.5 RNA extraction and illumina sequencing

Total RNA was extracted from pooled homogenates of six insects of each of the five strains detailed in this study using the Bioline Isolate RNA Mini Kit (Bioline, London, UK) according to the manufacturer's guidelines. Prior to the RNAseq experiment the quality and quantity of RNA was checked using a NanoDrop spectrophotometer (Nanodrop Technologies, Wilmington, DE, USA). Total RNA was used as a template for the generation of barcoded libraries (TrueSeq RNA library preparation, Illumina). Libraries were sequenced by The Genome Analysis Centre (TGAC, Norwich, UK) with replicates multiplexed for sequencing on an Illumina HiSeq 2500 flowcell (100 bp paired end reads) to generate at least 15 million reads per biological replicate. FastQC (version 0.11.2) was used to check the quality of the raw reads obtained.

### 2.6 SNP calling of RNA-Seq reads aligned to BPH *Rdl*

Raw reads of each BPH strain were mapped to the BPH *Rdl* reference gene sequence (accession no KX592155). Geneious R8's (Biomatters, Auckland, New Zealand) map to reference function was used with the BPH *Rdl* gene as the reference. Settings were: no gaps, maximum mismatches: 10%, minimum overlap identity: 80%, index word length: 14, maximum ambiguity: 4. All reads that aligned to AA residue 301 and 359 were then assessed for their nucleotide bases.

### 2.7 Pyrosequencing

**Fig. S1:**
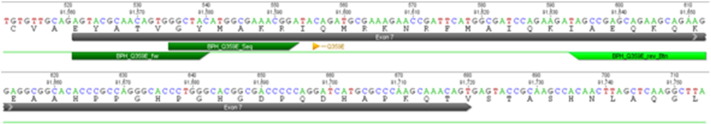
Partial nucleotide sequence of a 93 bp genomic fragment used for genotyping of the *Rdl* gene of *N. lugens*. The fragment shown includes the exon (marked in grey) carrying the mutation at position 359 (Q359, CAA or CAG and E359, GAG) (marked in yellow), flanking intron and primer sequences (in green).

For pyrosequencing purposes genomic DNA was extracted from individual BPH adults either using Microlysis-Plus-DNARelease Buffer (Microzone, UK) or QuickExtract Solution (Epicentre, USA) according to the supplier's recommended protocol. *Rdl* gene fragments were amplified by PCR from 50 ng aliquots of gDNA using two primers for the desired target sequence (Table S1; e.g. BPH_Q359E_fw & BPH_Q359E_rev_Btn for *Rdl* Q359E (Fig. S1) designed with Geneious 8 (Biomatters Ltd.) utilizing a partial sequence of the brown plant hopper GABA receptor gene. The pyrosequencing protocol comprised of 40 PCR cycles with 0.67 μM forward and reverse primer (one biotinylated, see Table S1) in 30 μl reaction mixture containing 2 × JumpStart Taq ReadyMix (Sigma-Aldrich, Germany) and cycling conditions of 95 °C for 3 min, followed by 40 cycles of 95 °C for 30 s, 57 °C for 30 s and 72 °C for 1 min, and a final incubation at 72 °C for 5 min in a C1000 Touch Thermal Cycler (Bio-Rad Laboratories, Inc.).

In addition, we also analysed the obtained plasmids for sequence correctness at the respective *Rdl* mutation positions by pyrosequencing, i.e. Q359E and A301S. A single PCR was conducted for each mutation site from 50 ng plasmid DNA using two primers (Q395E: BPH_Q359E_Plasmid_fw_Btn and BPH_Q359E_Plasmid_rev; A301S: BPH_A301S_Plasmid_fw_Btn and BPH_A301S_Plasmid_rev, Table S1). The PCR prior to pyrosequencing was carried out in 40 cycles with 0.5 μM forward and biotinylated reverse primer and 2 × JumpStart Taq ReadyMix in 30 μl reaction volume and cycling conditions of 95 °C for 3 min, followed by 40 cycles of 95 °C for 30 s, annealing temperature for Q359E PCR 52 °C and for A301S PCR 54 °C, followed by 72 °C for 1 min, and a final incubation at 72 °C for 5 min.

**Fig. S2:**
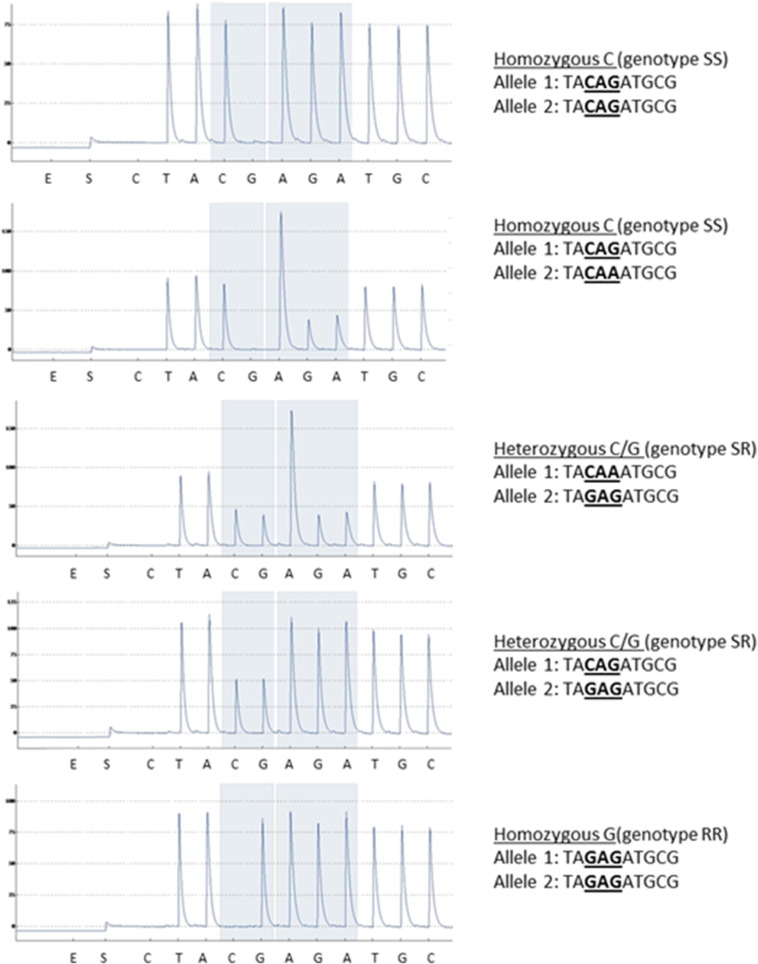
SNP-pyrosequencing assay results of the Q359E mutation in *N. lugens*. Grey bars and underlined nucleotides highlight the nucleotides of interest. Pyrograms are displaying homozygous SS, RR as well as heterozygous SR genotypes for the Q359E mutation found in *N. lugens* populations.

Single strand DNA preparation required for pyrosequencing was done using the PyroMark Q96 Vacuum Workstation (Qiagen) in combination with streptavidin coated beads (Streptavidin Sepharose) to separate the biotinylated strand of the PCR products. The pyrosequencing reactions were carried out according to the manufacturer's instructions utilizing the PyroMark Gold Q96 Reagent Kit (Qiagen) and the respective sequencing primers for genotyping (individual BPH adults: BPH_Q359E_seq; plasmids: BPH_Q359E_Plasmid_seq and BPH_A301S_Plasmid_seq; Table S1). The genotypes were analysed using the supplied PyroMark Q96 ID Software 2.5 (Qiagen). A typical example of the Q359E pyrosequencing results is shown (Fig. S2).

### 2.8 Preparation of cRNAs encoding *N. lugens Rdl* variants

Three variants of *N. lugens Rdl* were synthesized and sub-cloned into the expression vector pcDNA3.1(+) by Thermo Fisher Scientific (Life Technologies GmbH, Darmstadt, Germany): wildtype *Rdl* (accession no KX592155), *Rdl*-(A301S) and *Rdl*-(A301S + Q359E). The obtained plasmids were linearized by BbsI digestion according to manufacturer instructions (New England BioLabs Inc., USA), briefly: 20 μg plasmid DNA was incubated with 50 units BbsI for 3 h at 37 °C in a total volume of 100 μL. Subsequently the linearized DNA was purified using Qiagen QIAquick PCR Purification Kit (Qiagen GmbH, Germany). The capped cRNAs were generated using the mMESSAGE mMACHINE T7 transcription kit (ABI-Ambion, USA) and dissolved in RNase-free water at concentrations of 1718 ng/μL (wildtype *Rdl*), 1699 ng/μL (*Rdl*-(A301S)) and 1677 ng/μL (*Rdl*-(A301S + Q359E)).

### 2.9 *Rdl* expression and electrophysiological recordings in Xenopus oocytes

Defolliculated oocytes from *Xenopus laevis* in Barth's solution supplemented with gentamycin were received from Ecocyte Bioscience (Castrop-Rauxel, Germany). They were prepared and shipped one day before injection. Oocytes were injected with 75 nL of the prepared cRNA at a concentration of 100 ng/μL. All injections were performed using the automated injection system Roboinject (Multi Channel Systems MCS GmbH, Reutlingen, Germany). Oocytes were then incubated in Barth's solution with gentamycin (20 μg/mL) at 19 °C for 1–3 days. For electrophysiological measurements oocytes were superfused with Normal Frog Ringer (NFR) solution (DiacleanShop, Castrop-Rauxel, Germany) and voltage-clamped at a holding potential of − 80 mV using an automated two-electrode voltage clamp Roboocyte set-up (Multi Channel Systems MCS GmbH, Reutlingen, Germany). GABA was dissolved in NFR, whereas stock solutions of test compounds were prepared in DMSO and subsequently diluted with NFR to the desired test concentrations. Antagonist incubations were conducted with slight modifications according to Liu et al. (2015), i.e. antagonist solutions were perfused alone for 60 s after at least four successive GABA applications (EC_50_), followed by repeated antagonist/GABA (EC_50_) co-applications at 30 s intervals. Electrophysiological recordings were analysed using the software package Roboocyte V2.2.0 (Multi Channel Systems MCS GmbH, Reutlingen, Germany) and EC_50_/IC_50_-values were calculated from plotting normalized responses as a function of compound concentration using GraphPad Prism Software 5.03 (Graphpad Software, Inc., USA).

### 2.10 *D. melanogaster* insecticide bioassays

3–5 day old adult females were used in insecticide bioassays to assess the susceptibility of different fly strains to the technical compounds, ethiprole and fipronil (Sigma Aldrich, St. Louis, MO, USA). The flies were subjected to the insecticide in a contact/feeding bioassay. The full bioassay method is described in a previous paper [29]. The raw data was corrected for control mortality using Abbott's formula [30] and lethal concenctration values LC_50_ and LC_95_ were calculated by probit analysis using the GenStat® (2014, 17th Edition, ©VSN International Ltd., Hemel Hempstead, UK) statistical system.

### 2.11 Modelling

Protein modelling of the RDL *Nilaparvata* sequence was performed using the Orchestrar suite within the Certara software package Sybylx 2.1.1 (Certara L.P., St. Louis, MO). The crystal structure of the GluCl channel of *Caenorhabditis elegans* (PDB-Id: 3RHW) served as a template for the construction of the monomeric *Nilaparvata* homology model. Overall amino acid sequence identity between the monomers of the two species was 38.6%. The pentameric arrangement was realized by an iterative fit to each of the five subunits of the original crystal structure, followed by a subsequent energy minimization to remove any unwanted contacts and conformational distension from the complete model construction.

### 2.12 Database submission

Sequence data used in this study have been deposited at the National Center for Biotechnology Information as follows:

BioProject (accession no PRJNA331084).

BioSample (accession numbers SAMN05437238, SAMN05437239, SAMN05437240, SAMN05437241, SAMN05437242).

Run (accession no SRP079631).

## 3 Results

### 3.1 Fiprole bioassays

Diagnostic dose bioassays with ethiprole (Table 1), were performed on Nl33 and Nl55 soon after field collection, with high levels of resistance seen compared to the susceptible Bayer-S strain, previously reported in [5]. Log-dose probit-mortality data obtained from leaf dip bioassays are presented in detail (Table 2). Ethiprole resistance of the unselected Nl33 and Nl55 populations was 406-fold and 331-fold respectively compared to the lab susceptible strain Bayer-S. Selection of these strains with ethiprole (Nl33-eth and Nl55-eth) resulted in a drastic increase in resistance of > 14,000-fold. With a resistance ratio of 32-fold Nl33's level of resistance to fipronil was markedly lower compared with its ethiprole resistance. The same observation, but much more profound, was made for Nl55, which displays only a 3-fold resistance to fipronil. The two selected strains, in contrast, demonstrate similarly high levels of resistance to fipronil, with approximately 860-fold resistance against Bayer-S.

**Table 1:** Mortalities (%) (± standard error) for all *N. lugens* at two diagnostic doses (LD_95_ and 5 X LD_95_ of the susceptible strain) of ethiprole by leaf-dip bioassay.

Compound	Strain	3 mg L^− 1^ (± SE)	15 mg L^− 1^ (± SE)
Ethiprole	Bayer-S	100.00 ± nc	100 ± nc
	Nl33	6.72 (± 3.82)	nt
	Nl55	13.89 (± 5.69)	8.64 (± 4.68)

**Table 2:** Dose-response data for *N. lugens* laboratory susceptible and fiprol-resistant strains against ethiprole and fipronil applied as a leaf dip to adults.

Compound	Strain	Generations without selection	LC_50_ [mg L^− 1^]	95% limits	Slope (± SD)	RR
Ethiprole	Bayer-S	174	0.34	0.24–0.44	2.671 ± 0.432	1
	Nl33	27	138.3	90.82–198.3	1.32 ± 0.148	406.8
	Nl55	18	112.7	54.03–281.8	0.693 ± 0.124	331.5
	Nl33-eth	–	> 5000	–	–	> 14000
	Nl55-eth	–	> 5000	–	–	> 14000
Fipronil	Bayer-S	174	1.16	0.70–1.66	10.858 ± 0.864	1
	Nl33	46	37.13	1.06–137.3	1.259 ± 0.453	32
	Nl55	29	3.46	0.77–8.21	0.966 ± 0.197	3
	Nl33-eth	–	> 1000	–	–	> 860
	Nl55-eth	–	> 1000	–	–	> 860

### 3.2 Genotyping A301S and Q359E via sanger sequencing

**Fig. 1:**
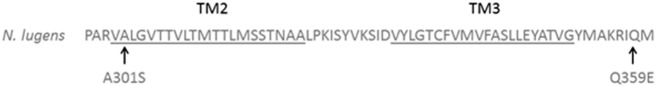
Amino acid sequence of TM2 and TM3 (TM regions underlined) from *N. lugens* RDL. The alanine and glutamine residues that are mutated in fiprole resistant strains are highlighted.

The mutations analysed in this study are shown (Fig.1). All strains were analysed for the presence of the A301S mutation by Sanger sequencing of an amplified 257 bp sequence from genomic DNA. Genotyping of A301S in Bayer-S confirmed the wild type genotype (Table 3). Nl55 on the other hand displayed a mix of genotypes, with only 12.5% of insects homozygous for A301S and 32.5% homozygous for the wildtype genotype. 100% of insects analysed from Nl55-eth carried the A301S mutation in the homozygous form. A novel mutation, Q359E, was also identified but only in Nl55 and Nl55-eth, with all other strains being 100% homozygous for the wildtype genotype at this AA residue. Nl55 displayed 7% of individuals homozygous for the Q359E mutation, with 57% of insects homozygous for the wildtype genotype. However, 87% of individuals were homozygous for the Q359E mutation in Nl55-eth, while the remaining 13% were heterozygous. Since the A301S mutation reached fixation in Nl55-eth it can be concluded that there are two A301S alleles present in that strain, one with and one without the Q359E mutation.

**Table 3:** Genotypes via Sanger sequencing of *N. lugens* strains for A301S and Q359E.

Population	A301S genotype (%)			Q359E genotype (%)		
	RR	SR	SS	RR	SR	SS
Bayer-S	0	0	100	0	0	100
Nl55	12.5	55	32.5	7.14	35.71	57.14
Nl55-eth	100	0	0	86.84	13.16	0

(Nl55 A301S N = 40, Nl55-eth A301S N = 40, Nl55 Q359E N = 28 and Nl55-eth Q359E N = 38).

### 3.3 SNP calling of A301S and Q359E via RNA-Seq

For all the strains, RNA-Seq reads were mapped against the BPH *Rdl* nucleotide sequence to observe any non-synonymous mutations, of which there were two: A301S and Q359E. Bayer-S SNP calling of A301S, displayed 100% of reads containing the wild type genotype at AA residue 301 (Table 4). Nl33 and Nl55 exhibited 85% and 80% of reads with the wild type genotype respectively. While for the Nl33-eth and Nl55-eth populations, 100% of reads contained the A301S mutation. In agreement with Sanger sequencing, the SNP calling of RNAseq data showed that the Q359E mutation was only found in Nl55 and Nl55-eth (Table 4), with 96% of Nl55-eth reads containing the Q359E mutation, compared to 27% for Nl55.

**Table 4:** SNP calling via RNA-Seq of *N. lugens* strains for A301S and Q359E.

		Nl33		Nl33-eth		Nl55		Nl55-eth	
		No. reads	%	No. reads	%	No. reads	%	No. reads	%
A301S	Total reads	20	–	34	–	10	–	18	–
	G (WT)	17	85	0	0	8	80	0	0
	T (Mut)	3	15	34	100	2	20	18	100
Q359E	Total reads	25	–	30	–	11	–	23	–
	C (WT)	25	100	30	100	8	72.73	1	4.35
	G (Mut)	0	0	0	0	3	27.27	22	95.65

### 3.4 Genotyping of Q359E via pyrosequencing

Ninety-six insects each of Nl55 and Nl55-eth were assessed for genotype at the 359 position (Table 5). Nl55 (unselected) showed 69% of individuals homozygous for Q, while only 3% homozygous for E, with the remaining individuals heterozygous for Q/E. Nl55-eth (selected) displayed 2% of individuals homozygous for Q, while 74% of individuals were homozygous for E, with the remaining individuals heterozygous for Q/E.

**Table 5:** Pyrosequencing of Q359E in two populations of *N. lugens.*

Population	Allele frequency		
	Q	Q/E	E
Nl55	0.69	0.28	0.03
Nl55-eth	0.02	0.24	0.74

### 3.5 Sensitivity of wildtype and mutant *N. lugens Rdl* receptors to GABA, ethiprole and fipronil

**Fig. 2:**
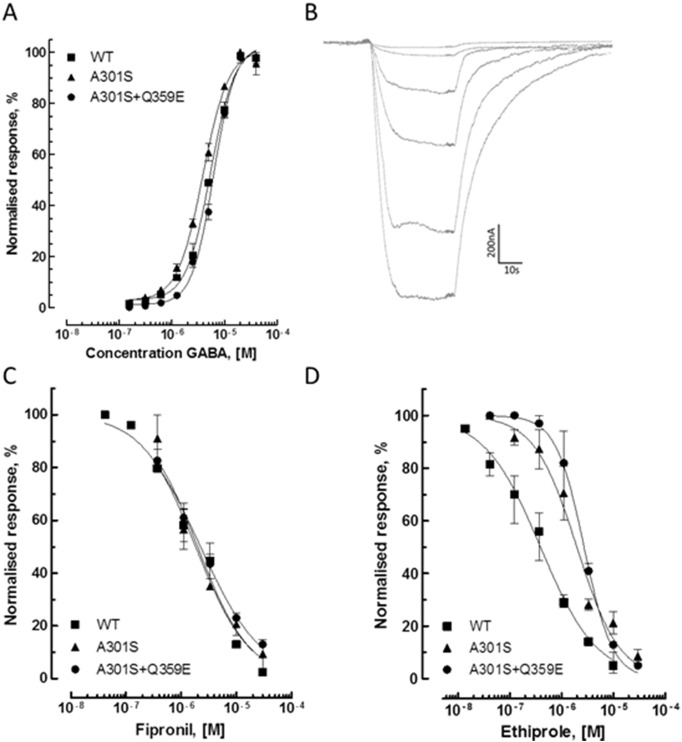
Effect of GABA and fiprole antagonists on GABA-induced currents in *N. lugens* RDL receptors functionally expressed in *Xenopus* oocytes. (A) GABA concentration-response curves on wildtype (WT) and mutant RDL variants carrying an A301S and A301S + Q359E amino acid substitution, respectively. Data are mean values ± SEM (n = 3); (B) Typical example of electrophysiological oocyte recordings showing the concentration-dependent action of GABA (10, 5, 2.5, 1.25, 0.625, 0.313 and 0.156 μM) on functionally expressed receptors (*Rdl* A301S). (C, D) Antagonist concentration-response curves for fipronil and ethiprole on three different RDL variants. The responses were normalized relative to the currents induced by 5 μM GABA for each receptor variant. Data are mean values ± SEM of 3–5 independent recordings.

All three cRNA variants resulted in functional GABA-gated chloride channels when injected in *Xenopus* oocytes, i.e. RDL wildtype (WT), RDL-(A301S) and RDL-(A301S + Q359E). Concentration dependent inward currents were obtained in voltage-clamp recordings in response to bath-applied GABA indicating the functional expression of homomeric RDL receptors in oocytes (Fig. 2A and B). The agonist pEC_50_-values calculated from the fitted curves were 5.43 ± 0.02, 3.90 ± 0.03 and 5.21 ± 0.02 for RDL wildtype, RDL-(A301S) and RDL-(A301S + Q359E), respectively. Both fipronil and ethiprole reduced the response of the three RDL subtypes to GABA (measured at EC_50_) in a concentration dependent manner (Fig. 2C and D). No significant difference in the antagonistic action of fipronil was measured between RDL subtypes: RDL wildtype, pIC_50_ 5.74 ± 0.06; RDL-(A301S), pIC_50_ 5.70 ± 0.08; and RDL-(A301S + Q359E), pIC_50_ 5.65 ± 0.08. However, for ethiprole significant differences in antagonistic action were obtained between RDL wildtype (pIC50 6.41 ± 0.05) and the two mutated subtypes, RDL-(A301S) (pIC_50_ 5.70 ± 0.06) and RDL-(A301S + Q359E) (pIC_50_ 5.56 ± 0.03).

### 3.6 *D. melanogaster* fiprole bioassays

The RDL-MD-RR (carrying *Rdl* A301S) strain displayed high levels of resistance to ethiprole with a resistance ratio > 4000 fold based on the LC_50_ when compared with the wildtype strain, Canton-S (Table 6). Against fipronil the RDL-MD-RR strain had a resistance ratio of only 6.9-fold.

**Table 6:** Log-dose probit mortality data for fiproles against *Drosophila melanogaster* strains.

							Resistance ratio	
Compound	Strain	LC_50_ [mg L^− 1^]	95% CL	LC_95_ [mg L^− 1^]	95% CL	Slope (± SD)	LC_50_	LC_95_
Ethiprole	Canton-S	5.73	4.77–6.77	22.39	17.14–33.08	2.777 ± 0.238	1	1
	RDL-MD-RR	> 25000	–	> 25000	–	–	> 4300	> 1100
Fipronil	Canton-S	1.27	0.77–1.85	9.04	5.29–25.55	1.931 ± 0.333	1	1
	RDL-MD-RR	8.82	5.34–13.7	62.36	33.11–238.1	1.936 ± 0.363	6.9	6.9

### 3.7 Synergist bioassays with PBO + fipronil

Synergistic bioassays were conducted with PBO on the highly fipronil resistant populations Nl33-eth and Nl55-eth to assess whether P450 monooxygenases (and esterases) could be potentially contributing to the resistance phenotype observed. Fipronil mortality of both populations was under 25% against a concentration of 500 mg L^− 1^ (Table 7) indicating that the majority of the individuals of both strains is unaffected by the application of PBO prior to exposure to fipronil.

**Table 7:** Mortalities (%) of ethiprole selected populations to fipronil after application of PBO.

Strain	100 mg L^− 1^	500 mg L^− 1^
Nl33-eth	20	16
Nl55-eth	10	24

## 4 Discussion

To date, the molecular basis of ethiprole resistance in *N. lugens* has remained unclear. A previous study linked esterase activity, and to a lesser extent P450s activity, to ethiprole resistance in *N. lugens* in central Thailand, based on the separate application of PBO, triphenyl phosphate and diethyl maleate as synergists prior to ethiprole exposure [27]. However, to date, no mutation(s) in the non-competitive antagonist binding site of RDL has been implicated in resistance to ethiprole. In the case of fipronil resistance, a potential novel mechanism of resistance was very recently implicated in a laboratory selected strain of *N. lugens* (see Introduction), but was not observed at sufficient frequency to cause resistance in field populations [24].

In this study, we identified two mutations in *Rdl* associated with phenylpyrazole resistance in two field strains. Both strains, Nl33 (Vietnam) and Nl55 (India) exhibited high levels of resistance to ethiprole, despite a long period of non-selection (27 and 18 generations respectively). When these strains were exposed to continuous ethiprole selection, their resistance markedly increased compared to the non-selected populations. We identified two mutations in these strains; the first was the previously reported A301S mutation [24], which was observed at low frequency in both parental field strains but rapidly rose in frequency and became fixed under ethiprole-selection. We further identified a novel mutation, Q359E, in one of the strains that also increased in frequency under selection. Subsequent functional analysis of the role of these mutations in resistance to fipronil and ethiprole, provided several lines of evidence to support a causal role of the A301S mutation in resistance to ethiprole.

Firstly, expression of recombinant wild-type and A301S RDL receptors in *Xenopus* oocytes followed by electrophysiological assays showed that presence of the A301S mutation reduces the sensitivity of the receptor to ethiprole 8–10-fold compared to wild-type providing strong evidence of a role in vitro. Further in vivo evidence of the role of this mutation in ethiprole resistance was also provided by insecticide bioassays of a *D. melanogaster* line with the same mutation, which exhibited 4000-fold resistance to ethiprole in comparison to a strain without the mutation.

In contrast to our findings with ethiprole very limited evidence was seen for a causal role of the A301S mutation in resistance to fipronil. In electrophysiological assays the recombinant A301S RDL receptor showed no significant shift in sensitivity to fipronil, with a response broadly similar to that of the wild type. A low level of resistance to fipronil was seen in the *D. melanogaster* line with the A301S mutation (around 7-fold compared to Canton-S). This result is similar to a recently reported study (13.8-fold) [16], on the same strain. A different strain of *D. melanogaster* strain carrying the same mutation was previously reported to show 73-fold resistance to fipronil [31], however, such high levels of resistance to fipronil were not apparent in our study or that carried out previously by Remnant et al. 2014.

As detailed above a second mutation, Q359E, was also observed in the Nl55 strain from India at low frequency but increased to high frequency upon ethiprole selection. All insects with Q359E carried it in combination with A301S. Since this mutation is never seen in isolation in the selected population, we decided to focus our analysis on the double mutant variant (A301S + Q359E) via electrophysiology to assess the effect of Q359E in tandem with A301S.

**Fig. 3:**
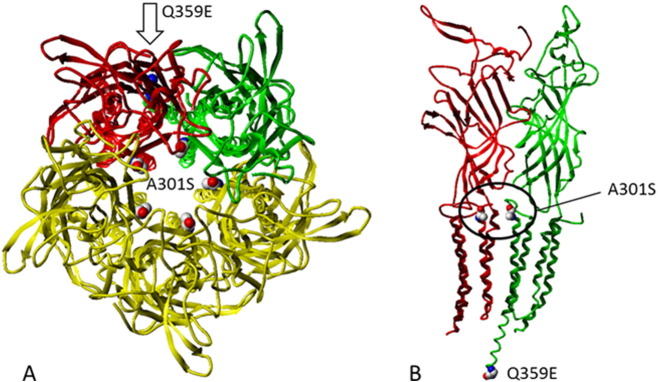
A) Top-view of the RDL GABA-R homo-pentamer (*Nl* RDL homology model based on 3RHW) showing three subunits in yellow, one in green and red, respectively. The mutation site A301S is located in the middle of the M2 transmembrane helices forming the channel pore. The other mutation site Q359E is located intracellularly at the end of helix M3 outside the pore region (indicated by an arrow). B) Side-view showing two of the RDL subunits and the location of the mutation site A301S in transmembrane pore helix M2, whereas mutation site Q359E is located > 40 Å from this residue (the helical structure of the domain is proposed as amino acid positions 337–428 are missing in the modelling template 3RHW).

Our data suggest that in comparison to A301S, the Q359E mutation plays no direct role in resistance to either ethiprole or fipronil with double mutant receptors displaying the same level of sensitivity to both compounds as the single mutant receptor (A301S). A model of the *N. lugens* RDL channel places the Q359E mutation at least > 40 Å away from the key A301S residue which could be a potential reason for its lack of direct impact (Fig. 3). However, a previous study using the Drosophila Genetic Reference Panel (DGRP) lines identified three fipronil resistant strains (A301S + T350S, A301S + T360I and A/S301 + M/I360) demonstrating the ability of multiple mutations in the *Rdl* to cause fipronil insensitivity [16]. Duplication at the *Rdl* locus has been described, and demonstrated the ability to accrue resistance mutations but maintain wildtype functionality in this insecticide target site [32]. In this study the Q359E mutation has only been tested in vitro and it would be interesting in future to examine its role in vivo in either *N. lugens* or *Drosophila* by using transgenic approaches such as the CRISPR/Cas system [29].

In the light of our results there are two possible explanations for the increase in frequency of the Q359E mutation under ethiprole selection. Firstly, it is a random polymorphism that because of its close proximity to A301S has hitchhiked to high frequency due to the physical linkage of the two mutations and the adaptive advantage of A301S. Secondly, this mutation, while not directly contributing to ethiprole resistance, may have a fitness benefit, to *N. lugens* individuals that carry this mutation in combination with A301S. For example, the Q359E mutation might act as a compensatory mutation for A301S as has been recently claimed for the R299Q substitution (see Introduction). Our results do not support this idea as recombinant receptors with A301S alone and A301S + Q359E show the same affinity for the native ligand GABA. Furthermore, A301S has been shown to persist in other insect species at high frequency in the absence of insecticide selection [33], suggesting it may have a minimal fitness penalty.

**Fig. S3:**
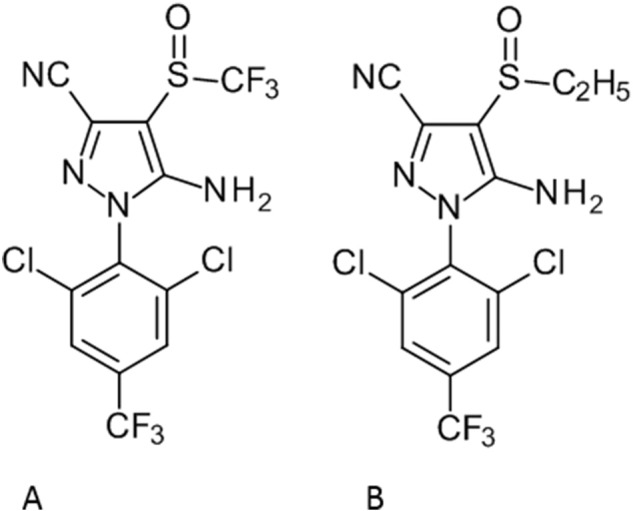
Chemical structure of A) Fipronil. B) Ethiprole.

The A301S mutation was one of the first target-site resistance mutations to be described in insects and has since appeared in a wide array of different insect species [32]. Originally described as the primary mechanisms of resistance to cyclodienes, it has also been linked with low level cross-resistance to fipronil [16], [17]. The effect of A301S in relation to cyclodiene resistance is two-fold, it reduces insecticide binding and destabilises the antagonist favoured structure of the RDL channel [34]. Surprisingly, this mutation has never been previously implicated in ethiprole resistance. Fipronil and ethiprole are highly structurally similar (Fig. S3) and so it is surprising that the A301S mutation can provide such effective resistance against ethiprole, but not to the same extent against fipronil.

The extremely high resistance levels seen in BPH strains selected with ethiprole, cannot be completely explained by the *Rdl* A301S mutation. The difference between wild-type and A301S RDL constructs in the voltage clamp recordings, was not enough to be wholly responsible for the resistance described in Table 2. Therefore, there must be another mechanism of resistance capable of causing resistance to ethiprole within the BPH populations tested here. We hypothesise that the unknown fipronil resistance mechanism (discussed later) could cause cross resistance to ethiprole, and therefore explain the very high levels of resistance in these BPH strains.

Zhang et al. recent study on R299Q and A301S mutations in RDL and their correlation with fipronil resistance [24], has similarities with our study. The finding of a novel mutation existing only in tandem with the A301S mutation is a key finding. However, the new mutation they describe (R299Q) appears to increase resistance to fipronil when combined with A301S, further than A301S by itself. Although we find a novel mutation, it does not have the same direct impact as R299Q. Similar to Zhang et al. we also find that the RDL mutations we analysed in this study are not the main mechanism of resistance to fipronil in the BPH populations tested.

The lack of impact of either of the RDL mutations against fipronil led us to carry out tests with the P450 and esterase inhibitor PBO to explore if these enzyme systems are involved in resistance to this compound. In this regard, recent research has used the same approach to implicate metabolic mechanisms in resistance to fipronil in *N. lugens* [24]. In our study the application of PBO had no noticeable impact on the fipronil resistance of the resistant populations, Nl33-eth and Nl55-eth suggesting P450s and/or esterases are either not involved in resistance or play a minor role. However, in contrast to our study Zhang et al. applied a mixture of synergists (PBO, triphenyl phosphate and diethyl maleate), so it is possible that other enzyme systems that are inhibited by triphenyl phosphate and/or diethyl maleate are involved in resistance such as glutathione S-transferases [24].

An interesting observation from our selection experiments was that the selection of the Nl33 and Nl55 strains with ethiprole also increased resistance to fipronil. Despite this observation, our data clearly suggest that the mutations analysed in this study are not the explanation for the increased fipronil resistance in the Nl33-eth and Nl55-eth strains and the molecular basis of fipronil resistance in these strains requires further investigation. However, this finding is of field relevance as it suggests that fipronil would not be a viable substitute for ethiprole, if ethiprole was no longer able to control *N. lugens* within economic thresholds.

In conclusion, two mutations (A301S and Q359E) were identified in the *Rdl* gene of *N. lugens* and assessed for their potential role in resistance to fiproles. Our results indicate that the common A301S mutation confers resistance to ethiprole, a widely used insecticide for the control of brown planthopper. However, neither this mutation nor the novel mutation Q359E causes significant resistance to fipronil based on the in vitro and in vivo studies conducted here. Our finding that selection with ethiprole also selects for cross-resistance to fipronil is relevant to the future application of these insecticides in the field and the design and implementation of resistance management programmes.

The following are the supplementary data related to this article.

Table S1 Primer sequences used for Sanger sequencing and genotyping by pyrosequencing. Table S1

## Funding

This work was funded by Bayer CropScience (S2088). Rothamsted Research receives grant-aided support from the Biotechnology and Biosciences Research Council of the United Kingdom (BBS/E/C/00005193).

## References

[1] Cabauatan P.Q., Cabunagan R.C., Choi I.-R., Heong K.L., Hardy B. (2009). Rice viruses transmitted by the brown planthopper *Nilaparvata lugens* Stål. Planthoppers: New Threats to the Sustainability of Intensive Rice Production Systems in Asia.

[2] Cheng J., Heong K.L., Hardy B. (2009). Rice planthopper problems and relevant causes in China. Planthoppers: New Threats to the Sustainability of Intensive Rice Production Systems in Asia.

[3] Hemingway J., Karunaratne S., Claridge M.F. (1999). Insecticide resistance spectrum and underlying resistance mechanisms in tropical populations of the brown planthopper (*Nilaparvata lugens*) collected from rice and the wild grass *Leersia hexandra*. Int. J. Pest Manage..

[4] Nagata T., Kamimuro T., Wang Y.C., Han S.G., Noor N.M. (2002). Recent status of insecticide resistance of long-distance migrating rice planthoppers monitored in Japan, China and Malaysia. J. Asia Pac. Entomol..

[5] Garrood W.T., Zimmer C.T., Gorman K.J., Nauen R., Bass C., Davies T.G.E. (2016). Field-evolved resistance to imidacloprid and ethiprole in populations of brown planthopper *Nilaparvata lugens* collected from across south and east Asia. Pest Manag. Sci..

[6] Matsumura M., Sanada-Morimura S. (2010). Recent status of insecticide resistance in Asian rice planthoppers. JARQ (Jpn Agric Res Q).

[7] Zhang X.L., Liu X.Y., Zhu F.X., Li J.H., You H., Lu P. (2014). Field evolution of insecticide resistance in the brown planthopper (*Nilaparvata lugens* Stal) in China. Crop. Prot..

[8] Cole L.M., Nicholson R.A., Casida J.E. (1993). Action of phenylpyrazole insecticides at the gaba-gated chloride channel. Pestic. Biochem. Physiol..

[9] Bloomquist J.R., Ishaaya I. (2001). GABA and glutamate receptors as biochemical sites for insecticide action. Biochemical Sites of Insecticide Action and Resistance.

[10] Knipple D.C., Soderlund D.M. (2010). The ligand-gated chloride channel gene family of *Drosophila melanogaster*. Pestic. Biochem. Physiol..

[11] Sparks T.C., Nauen R. (2015). IRAC: mode of action classification and insecticide resistance management. Pestic. Biochem. Physiol..

[12] Casida J.E., Durkin K.A. (2013). Neuroactive insecticides: targets, selectivity, resistance, and secondary effects. Annu. Rev. Entomol..

[13] Caboni P., Sammelson R.E., Casida J.E. (2003). Phenylpyrazole insecticide photochemistry, metabolism, and GABAergic action: ethiprole compared with fipronil. J. Agric. Food Chem..

[14] R.H. ffrench-Constant (2013). The molecular genetics of insecticide resistance. Genetics.

[15] R.H. ffrench-Constant, Roush R.T., Mortlock D., Dively G.P. (1990). Isolation of dieldrin resistance from field populations of *Drosophila melanogaster* (Diptera: Drosophilidae). J. Econ. Entomol..

[16] Remnant E.J., Morton C.J., Daborn P.J., Lumb C., Yang Y.T., Ng H.L., Parker M.W., Batterham P. (2014). The role of *Rdl* in resistance to phenylpyrazoles in *Drosophila melanogaster*. Insect Biochem. Mol. Biol..

[17] Bass C., Schroeder I., Turberg A., Field L.M., Williamson M.S. (2004). Identification of the *Rdl* mutation in laboratory and field strains of the cat flea, *Ctenocephalides felis* (Siphonaptera: Pulicidae). Pest Manag. Sci..

[18] Le Goff G., Hamon A., Berge J.B., Amichot M. (2005). Resistance to fipronil in *Drosophila simulans*: influence of two point mutations in the RDL GABA receptor subunit. J. Neurochem..

[19] Nakao T., Naoi A., Kawahara N., Hirase K. (2010). Mutation of the GABA receptor associated with fipronil resistance in the whitebacked planthopper, *Sogatella furcifera*. Pestic. Biochem. Physiol..

[20] Nakao T., Kawase A., Kinoshita A., Abe R., Hama M., Kawahara N., Hirase K. (2011). The A2'N mutation of the RDL gamma-aminobutyric acid receptor conferring fipronil resistance in *Laodelphax striatellus* (Hemiptera: Delphacidae). J. Econ. Entomol..

[21] Nakao T., Hama M., Kawahara N., Hirase K. (2012). Fipronil resistance in *Sogatella furcifera*: molecular cloning and functional expression of wild-type and mutant RDL GABA receptor subunits. J. Pestic. Sci..

[22] Wolff M.A., Wingate V.P.M. (1998). Characterization and comparative pharmacological studies of a functional y-aminobutyric acid (GABA) receptor cloned from the tobacco budworm, *Heliothis virescens* (Noctuidae: Lepidoptera) Invertebr. Neuroscience.

[23] Lees K., Musgaard M., Suwanmanee S., Buckingham S.D., Biggin P., Sattelle D. (2014). Actions of agonists, fipronil and Ivermectin on the predominant in vivo splice and edit variant (RDL_bd_, I/V) of the *Drosophila* GABA receptor expressed in *Xenopus laevis* oocytes. PLoS One.

[24] Zhang Y., Meng X., Yang Y., Li H., Wang X., Yang B., Zhang J., Li C., Millar N.S., Liu Z. (2016). Synergistic and compensatory effects of two point mutations conferring target-site resistance to fipronil in the insect GABA receptor RDL. Sci. Rep..

[25] Sahaya S., Ek-amnuay J., Wongnikong W., Angmanee P., Pechthamros S., Chamroenma K. (2010). Efficacy of some insecticides against brown planthopper, *Nilaparvata lugens* (Stål) on rice field. Annual report of the Entomology and Zoology Group.

[26] Bayer CropScience (2007). Curbix: the long-lasting solution against brown planthoppers. Courrier.

[27] Punyawattoe P., Han Z.J., Sriratanasak W., Arunmit S., Chaiwong J., Bullangpoti V. (2013). Ethiprole resistance in *Nilaparvata lugens* (Hemiptera: Delphacidae): possible mechanisms and cross-resistance. Appl. Entomol. Zool..

[28] Elzaki M.E., Zhang W., Han Z. (2015). Cytochrome P450 CYP4DE1 and CYP6CW3v2 contribute to ethiprole resistance in *Laodelphax striatellus* (fallen). Insect Mol. Biol..

[29] Zimmer C.T., Garrood W.T., Puinean A.M., Eckel-Zimmer M., Williamson M.S., Davies T.G.E., Bass C. (2016). A CRISPR/Cas9 mediated point mutation in the alpha 6 subunit of the nicotinic acetylcholine receptor confers resistance to spinosad in *Drosophila melanogaster*. Insect Biochem. Mol. Biol..

[30] Abbott W.S. (1925). A method of computing the effectiveness of an insecticide. J. Econ. Entomol..

[31] Cole L.M., Roush R.T., Casida J.E. (1995). *Drosophila* GABA-gated chloride channel: modified [^3^*H*]EBOB binding site associated with Ala → Ser or Gly mutants of *Rdl* subunit. Life Sci..

[32] Remnant E.J., Good R.T., Schmidt J.M., Lumb C., Robin C., Daborn P.J., Batterham P. (2013). Gene duplication in the major insecticide target site, *Rdl*, in *Drosophila melanogaster*. Proc. Natl. Acad. Sci. U. S. A..

[33] Thompson M., Steichen J.C., R.H. ffrench-Constant (1993). Conservation of cyclodiene insecticide resistance-associated mutations in insects. Insect Mol. Biol..

[34] Zhang H.G., ffrench-Constant R.H., Jackson M.B. (1994). A unique amino acid of the *Drosophila* GABA receptor with influence on drug sensitivity by two mechanisms. J. Physiol..

