# Supplementary material for: Influence of the RDL A301S mutation in the brown planthopper Nilaparvata lugens on the activity of phenylpyrazole insecticides
Source: Pestic Biochem Physiol. 2017 Oct;142:1–8. doi: 10.1016/j.pestbp.2017.01.007 (PMC5672059; doi:10.1016/j.pestbp.2017.01.007)
Supplement: Table S1 — Primer sequences used for Sanger sequencing and genotyping by pyrosequencing. [file mmc1.docx]

**Table S1** Primer sequences used for Sanger sequencing and genotyping by pyrosequencing

| Primer name | Sequence 5´to 3´ |
| --- | --- |
| BPH_A301S_Sanger_fw  BPH_A301S_Sanger_rev  BPH_Q359E_Sanger_rev  BPH_Q359E_fw  BPH_Q359E_rev_Btn  BPH_Q359E_seq  BPH_Q359E_Plasmid_fw_Btn  BPH_Q359E_Plasmid_rev  BPH_Q359E_Plasmid_seq  BPH_A301S_Plasmid_fw_Btn  BPH_A301S_Plasmid_rev  BPH_A301S_Plasmid_seq | ATCCAGTTCGTGCGTTCGATG  AGCAACGACGCGAACACCAT  TCCGAAAGCGCTCTACATGA  AGTACGCAACAGTGGGCTAC  [btn]CTTCTGCTTCTGCTCGGCTA  GGCTACATGGCGAAACGGA  [btn]CCTGTTTCGTGATGGTGTTCGC  GCTTCTGCTCGGCGATCTTCTG  CCATGAACCGGTTCTTCCGC  [btn]CCAGCGGCCTGATCGTGATTATC  GCTCATCAGGGTGGTCATGGTC  TCAGCACGGTGGTCACKCC |
